# Ancient DNA sheds light on the ancestry of pre-hispanic Canarian pigs

**DOI:** 10.1186/s12711-015-0115-7

**Published:** 2015-05-06

**Authors:** Iñigo Olalde, Juan Capote, María C Del-Arco, Pablo Atoche, Teresa Delgado, Rafael González-Anton, Jorge Pais, Marcel Amills, Carles Lalueza-Fox, Oscar Ramírez

**Affiliations:** Institut de Biologia Evolutiva (CSIC - Universitat Pompeu Fabra), Barcelona, Spain; Instituto Canario de Investigaciones Agrarias, La Laguna, Tenerife, Spain; Departamento de Geografía e Historia, Universidad de La Laguna, La Laguna, Tenerife, Spain; Departamento de Ciencias Históricas, Universidad de Las Palmas de Gran Canaria, Las Palmas de Gran Canaria, Spain; Museo Canario, Las Palmas de Gran Canaria, Spain; Museo de la Naturaleza y el Hombre-M Arqueológico, Tenerife, Spain; Museo Arqueológico Benahorita, La Palma, Spain; Center for Research in Agricultural Genomics (CSIC-IRTA-UAB-UB), Campus UAB, 08193 Bellaterra, Spain

## Abstract

**Background:**

Canarian Black (CB) pigs belong to an autochthonous and endangered breed, which is spread throughout the Canarian archipelago. It is commonly accepted that they represent a relic of the pig populations that were bred by the Berbers in North Africa over millennia. It is important to note that the geographic isolation of the Canary Islands has preserved this genetic legacy intact from foreign introgressions until the Spanish conquest of the archipelago in the 15^th^ century. Ten years ago, it was demonstrated that, in CB pigs, the frequency of the Asian A2 cytochrome-b haplogroup reached 73%. The current work aimed at investigating whether this observation is explained by either a recent or an ancient introgression of CB pigs with Far Eastern pigs.

**Results:**

Genetic analyses of 23 ancient samples from pre-hispanic Canarian pigs (420 to 2500 years before present) showed that Near Eastern and Far Eastern genetic signatures were totally absent in the primitive Canarian pre-hispanic pigs. Indeed, the haplotypes detected in these pigs were closely related to those of North African and European wild boars.

**Conclusions:**

Our results demonstrate that the high frequency of the Far Eastern mitochondrial cytochrome B A2 haplotype in modern Canarian Black pigs probably corresponds to a relatively recent introgression with British breeds.

**Electronic supplementary material:**

The online version of this article (doi:10.1186/s12711-015-0115-7) contains supplementary material, which is available to authorized users.

## Findings

The only living representative of the domestic swine that was introduced into the Canary Islands 3000 YBP (years before present) is the Canarian Black (CB) pig, which in the 1980’s was very near extinction. However, an active conservation program has allowed the population to reach a census of a few hundred individuals [1,2]. Analysis of mitochondrial genome variation in modern CB pigs revealed that the Far Eastern A2 cytochrome-b (*MT-CYB*) haplogroup reached a high frequency in this breed (up to 73%) [3]. Far Eastern haplotypes are absent in local European breeds, such as the Iberian, Mangalitza, Majorcan Black or Basque pigs [4]. Clop et al. [3] proposed two alternative explanations for the presence of Far Eastern haplotypes in the mitochondrial genome of CB pigs *i.e.* either introgression of CB pigs with improved British breeds that had been extensively hybridized with Chinese sows in the 18^th^ and 19^th^ centuries to select for pigs with earlier reproductive maturity and increased fatness [5], or conversely, a more ancient introduction as a consequence of the settlement of Berber tribes in the Canary Islands. Although this latter interpretation is less likely, Far Eastern haplotypes are very common in East African breeds such as the Mukota breed [4] and it is possible that these haplotypes may have diffused westwards, as is the case for indicine alleles in cattle [6].

**Figure 1:**
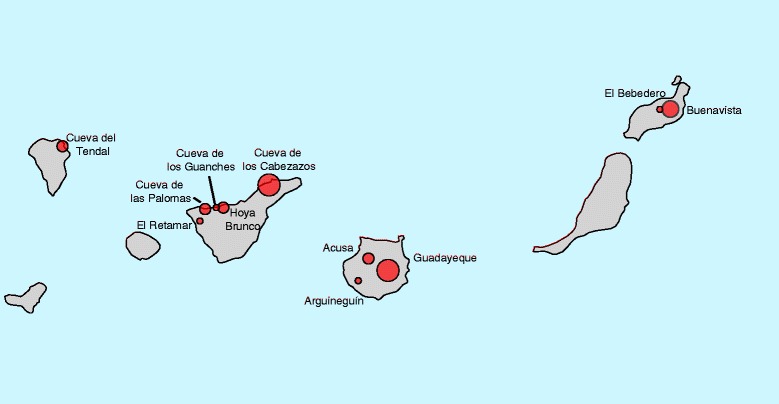
**Geographic locations of the archaeological assemblies from where ancient samples were collected.** The size of the circles is proportional to the number of samples analysed.

Obviously, these two alternative scenarios cannot be ascertained with mitochondrial data from modern CB samples. Thus, we decided to survey the mitochondrial variation of 23 ancient pig samples that cover a large period of time (from ≈ 420 to 2500 YBP) and represent 11 pre-Hispanic archeological sites across four of the seven Canary Islands (Figure 1) and [see Additional file 1; Additional file 2: Table S1]. Total DNA was extracted in laboratories that are dedicated to the analysis of ancient DNA at the Institute of Evolutionary Biology and University of Pompeu Fabra in Barcelona by applying proteinase-K digestion followed by phenol-chloroform precipitation and a column concentration (Amicon), as described elsewhere [7]. Extracts from skin samples were subsequently purified with a gene clean silica method using a DNA extraction Kit (Fermentas, USA). To the best of our knowledge, no previous work on modern pigs had ever been conducted at this laboratory and standard precautions for experiments involving ancient DNA samples were followed (see Appendix).

Pig specific primers [see Additional file 2: Table S2]) were designed to amplify two non-overlapping sequences of 89 (PCR1) and 77 bp (PCR2) corresponding to the *MT-CYB* gene. Sequence PCR1 contains the diagnostic single nucleotide polymorphisms (SNPs) that are located at positions 15036, 15038, 15041, 15044 and 15045 and differentiate European (E1, E2, E3 and E4) from Asian (A1, A2, A3, A4) haplogroups [3,8] and sequence PCR2 contains two SNPs that discriminate between Near Eastern *vs* Western (Europe and North Africa) haplotypes [4]. Each fragment was amplified using a two-step PCR protocol [9]. Amplified products were purified with a gene clean silica method using the DNA Extraction Kit (Fermentas, USA) and cloned using the Topo TA cloning kit (Invitrogen, The Netherlands). White colonies were subjected to 30 cycles of PCR with M13 universal primers and subsequently sequenced with an Applied BioSystems 3100 DNA sequencer, at the *Servei de Seqüenciació* of the *Universitat Pompeu Fabra* (Barcelona).

Amplicon sequences of PCR1 and PCR2 were obtained from 21 of 23 and 8 of 10 specimens of pre-Hispanic Canary pigs, respectively. PCR1 was analyzed in all 23 ancient pig samples collected. For PCR2, we excluded the samples for which PCR1 was not amplified and also most of the samples of mummified pig skin because, after purification and amplifications, the amount of DNA extracted was not sufficient [see Additional file 2: Table S1]. Although the high temperatures of the climatic conditions of the Canary Islands do not favor DNA preservation, the success rates of amplification (91.3% and 80%, respectively) were very high [10,11]. For identical temperature and environmental settings, preservation of ancient DNA is highly correlated with sample age [12]. Two of the samples for which no successful amplification was achieved (Buenavista 3 and Lanzarote 12, both from Lanzarote) were among the four oldest materials in the assemblage. The third sample for which amplification failed (Guadayaque11/3) originated from a leather skin that was used as shroud on the mummies. Possibly, the presence of inhibitor substances used during the embalming process may have prevented DNA amplification.

**Figure 2:**
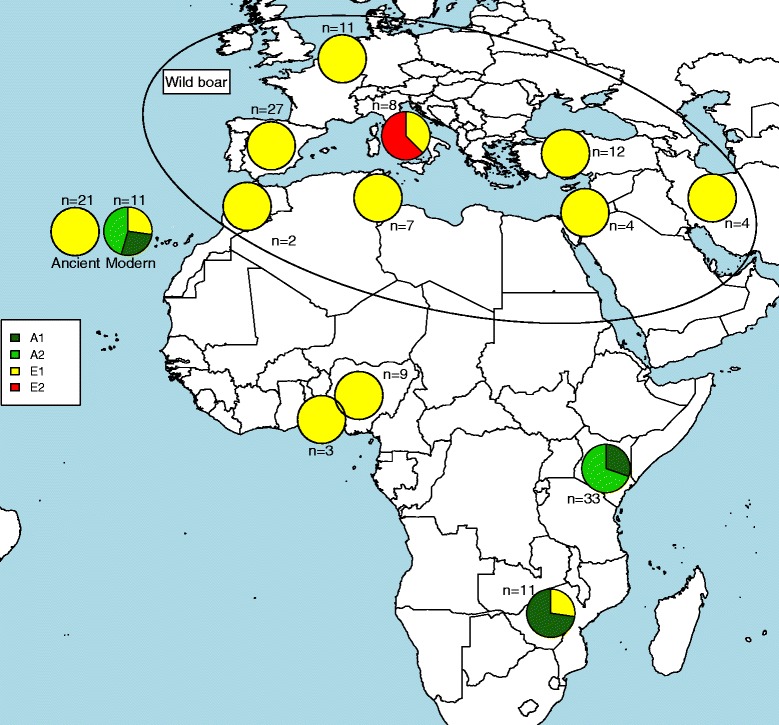
**Comparison of frequencies of mitochondrial** ***MT-CYB*** **haplogroups in the ancient Canarian pigs (described in this study) and modern pigs from Africa and the Canary Islands and wild boar from Near East and Europe described previously by Ramirez et al. [** 4 **].**

All PCR1 DNA sequences obtained from the 21 ancient pig samples from the Canary Islands belonged to a single haplogroup, *i.e. MT-CYB* haplogroup E1 (Figure 2) and [see Additional file 2: Table S1, Additional file 3: Figure S1]. Given that haplogroup E1 is represented by distinct haplotypes that segregate in wild boars from the Near East, Europe and North Africa, it does not allow us to conclude on the geographical origin of the primitive Canarian pre-hispanic pigs. Interestingly, the eight ancient pig samples that provided PCR2 amplicons harboured Western haplotypes [see Additional file 3: Figure S2]. It is important to note that Western *MT-CYB* variants have negligible frequencies in Near Eastern wild boars and they probably reflect introgression with Western pigs or feralization of domestic pigs [4,13]. This result agrees well with data on the autosomal nuclear genome: based on 60K SNP genotypes of Near Eastern and European pigs and wild boars, Manunza et al. [14] demonstrated that there was no Near Eastern footprint in extant Canarian pigs. The absence of such signatures in the eight pre-hispanic Canarian samples that varied in age from ≈ 960 to 2500 years suggests that, at the beginning of the first millennium BC, the Near East signature was absent, or at low frequency, in domestic pig populations from western North Africa.

Our data not only provide a first glimpse on the mitochondrial gene pool of pigs that probably have a Berber ancestry, but also help to solve a puzzling finding reported by Clop et al. [3] 10 years ago *i.e.* the high frequency of the Far Eastern A2 *MT-CYB* haplotype in CB pigs. Our results on ancient DNA strongly support the former hypothesis i.e. the complete absence of Far Eastern haplotypes in the dataset of ancient pig samples (from 11 locations across four of the seven Canary Islands) suggests that native CB pigs did not carry Far Eastern alleles. According to García-Martín and Capote [2], pigmentation and ear morphology and size of Canarian pigs resemble those observed in the Berkshire pig breed. Interestingly, the A2 haplotype segregates at high frequencies (around 35%) in this British local breed [8], which provides evidence for a recent Berkshire introduction. From an historical point of view, crossbreeding of CB pigs with British and Iberian breeds has been widely documented [15,16].

Certain insights about the origin of modern domestic breeds can only be obtained through the analysis of ancient DNA [13,17–19]. In this study, partial sequencing of the *MT-CYB* sequence in pig samples from Canarian archaeological assemblages has allowed us to demonstrate that the presence of Asian *MT-CYB* alleles in CB pigs was the result of a recent introgression event (probably with British breeds). However, ascertainment bias is a problem for proper interpretation of results that are exclusively based on maternal data. Analyses of ancient samples based on whole-genome sequencing may contribute to better understand the process of domestication and breed formation.

## Additional files

Additional file 1:
**Sample information.** Description of the pig ancient samples that were used in this study and collected at different sites: Tenerife [24–29], Gran Canaria [30] and Lanzarote [31–36]. Additional file 2: Table S1. Archaeological sites and age of Canarian pig samples used. This Table provides the ID, lab codes, archeological site and age of the Canarian pig ancient samples from which cytochrome B haplogroups (Fragment 1) and haplotypes (Fragment 2) were retrieved. List of primers used for amplification of the two 89 and 77 bp sequences of the *MT-CYB* gene. **Table S2.** provides the names and sequences of the primers used to amplify the two 89 and 77 bp sequences of the *MT-CYB* gene. Primers were designed with the Primer 3 software (http://bioinfo.ut.ee/primer3-0.4.0/). Additional file 3: Figure S1. Alignment of the 48-bp sequences (PCR1) of the *MT-CYB* gene fragment 1 obtained from DNA from 21 Canarian pig ancient samples. Description: The diagnostic SNPs that are located at positions 15036, 15038, 15041, 15044 and 15045 (indicated in red) differentiate European: E1 (GenBankID: KJ746666), E2 (EU531827) and E4 (GU211924) haplogroups and, also, Far Asian: A1 (KP257599), A2 (KM215171), A3 (AB015072), A4 (KJ746664) and A5 (GU135825) haplogroups. **Figure S2.** Alignment of the 37-bp sequences (PCR2) of the *MT-CYB* gene fragment 1 obtained from DNA from 8 Canarian pig ancient samples. Description: The two diagnostic SNPs (indicated in red) differentiate haplotypes of haplogroup E1 present in wild boar individuals from the Near East: H12 (GenBank ID: EU531818), H31 (EU531827), H52 (EU531832), H53 (EU531833), and H54 (EU531834); and from Europe: H1 (AY237496), H4 (AY237512), H15 (AB015072), H16 (EU531821), H17 (EF061501), H18 (AY237516), H24 (AF136542), H27 (EU531824), H32 (EU531828), H34 (EU531830), H65 (AM492593) and H66 (AM492620).

## Appendix

### Sequencing of mtDNA

DNA was extracted following the method described in detail in [20]. Extraction procedures were performed in an isolated pre-PCR area, by adopting the standard precautions for ancient DNA studies [21,22]. Multiple extraction and amplification negative controls to monitor for contamination in the reagents were added to each PCR reaction. During the whole study, no amplification products were obtained in these blank PCR controls.

Amplification was carried out according to a two-step PCR protocol reported by [9]. Both PCR steps were carried out with 2 units AmpliTaq Gold (ABI, USA), 1X AmpliTaq Gold buffer (ABI, USA), 2.5 mM MgCl_2_ (ABI, USA), and 500 μM of each dNTP. In the first multiplex step, 150 nM of each primer pair and 5 μl of DNA extract were added to a final reaction volume of 20 μl. The first amplification step consisted in a 12 min activation step at 94°C, followed by 27 cycles at 94°C for 20 s, 50°C for 20 s, and 72°C for 20 s. Five μl of a 1 to 10 dilution of the primary amplification product were used as template for the second PCR step. Conditions were the same as for the first step, except that the standard primer concentration was increased to 1.5 μM and that 33 cycles were performed, followed by a final step of 12 min at 72°C. Blank and mock PCR controls were included in each amplification step to monitor against contamination.

PCR products were visualized under UV light and the appropriate bands were excised from a low-melting point agarose gel and purified with a silica-based method. Subsequently, the amplification products were cloned in bacteria (TOPO-TA cloning kit, Invitrogen). Resulting white colonies were picked, amplified with M13 universal primers and sequenced with an ABI3730 capillary sequencer (Applied Biosystems).

We followed the criteria for authenticity described by Gilbert et al. [23]: (i) amplifications of both mtDNA sequences of the Buenavista1 sample were replicated in an independent laboratory (at the Universitat Pompeu Fabra); (ii) for all the samples, mock extractions and PCR blank controls were carried out and there was no evidence of contamination; and (iii) about 30% of the fragments were replicated twice [see Additional file 3: Figures S1 and S2].

To assign the genotype of the diagnostic SNPs, we used the majority rules consensus. If, for one or a few clones, additional substitutions are found present in the sequence of one PCR but not in the sequence of another PCR from the same sample, it is reasonable to attribute them to DNA damage, and thus they were not considered in the analyses.
