# Supplementary material for: Ancient DNA sheds light on the ancestry of pre-hispanic Canarian pigs
Source: Genet Sel Evol. 2015 May 6;47(1):40. doi: 10.1186/s12711-015-0115-7 (PMC4421913; doi:10.1186/s12711-015-0115-7)
Supplement: Additional file 1: — Sample information. Description of the pig ancient samples that were used in this study and collected at different sites: Tenerife [24–29], Gran Canaria [30] and Lanzarote [31–36]. [file 12711_2015_115_MOESM1_ESM.pdf]

Sample information

Tenerife

The sites from where the Tenerife samples were taken correspond to three habitation and two burial caves. Two of the habitations are located in medium-altitude mountains, La Cueva de las Palomas (Icod de los Vinos) [24,25] and La Cueva de Los Cabezazos (Tegueste) [26,27] and the third habitation is at La Cueva de Los Guanches [28,25] on a coastal cliff (Icod de los Vinos). At all these sites, continuous occupation is observed *i.e.* Los Guanches (from the mid IX century BC to the VI century AD), Las Palomas (from the III century BC till the Hispanic conquest) and Los Cabezazos (from the VI century AD till the Hispanic conquest). Food remains were sampled at all these sites.

In addition, samples were taken from the leather skins used as shrouds on the mummies that were found at two burial caves, *i.e.* El Retamar-Ucazme (Adeje), with varied funerary rituals (mummies, disarticulated bodies and ocre unction) [29] with 14C dating indicating a period around 1450 AD, and Hoya Brunco (La Guancha), which was occupied as a burial site during at least two centuries, with 14C dating on mummified remains indicating periods around 1050 AD and 1260 AD.

Several photos of samples from Tenerife are provided:

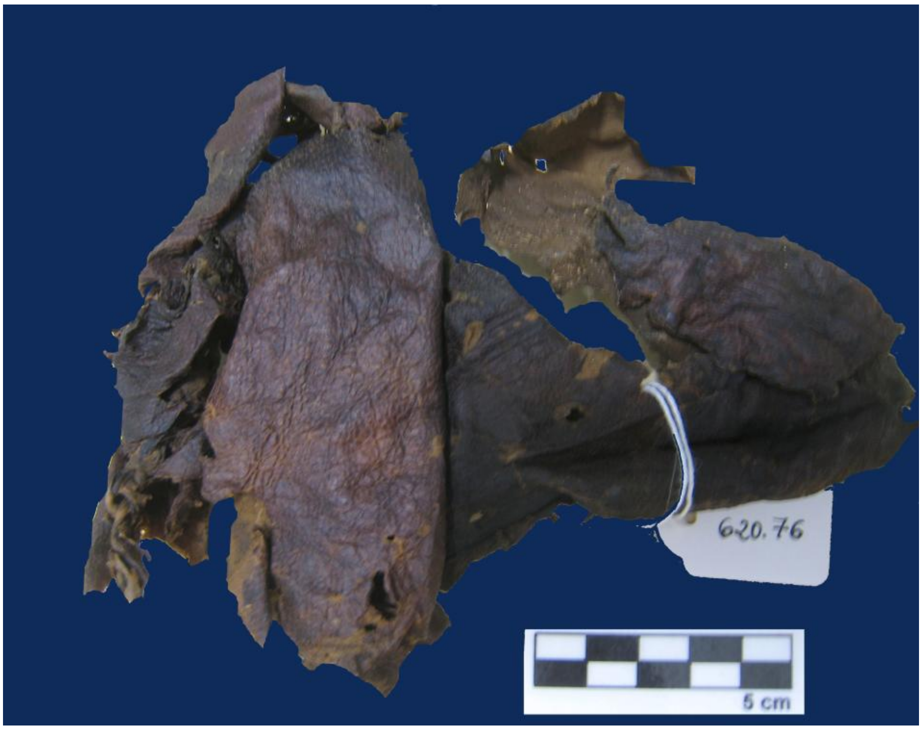

Photo 1. Sample LaGuancha1, skin of *Sus scrofa* used as shrouds on the mummies found at the Hoya Brunco burial cave (La Guancha, Tenerife) (©C. del Arco).

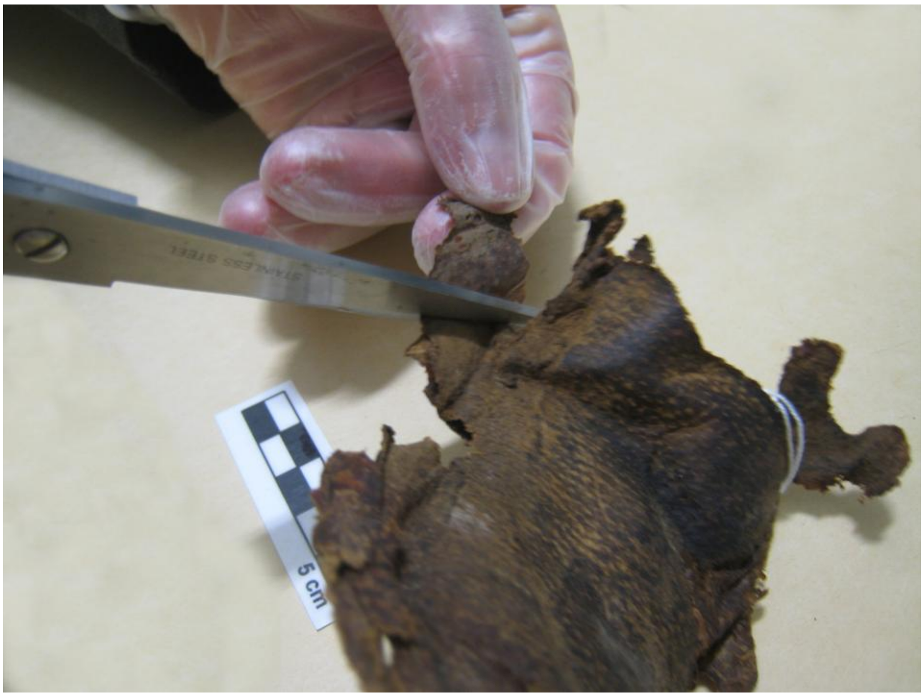

Photo 2. Taking a fragment (sample LaGuancha3) from skin of *Sus scrofa* used as shrouds on the mummies found at the Hoya Brunco burial cave (La Guancha, Tenerife) (© C. del Arco).

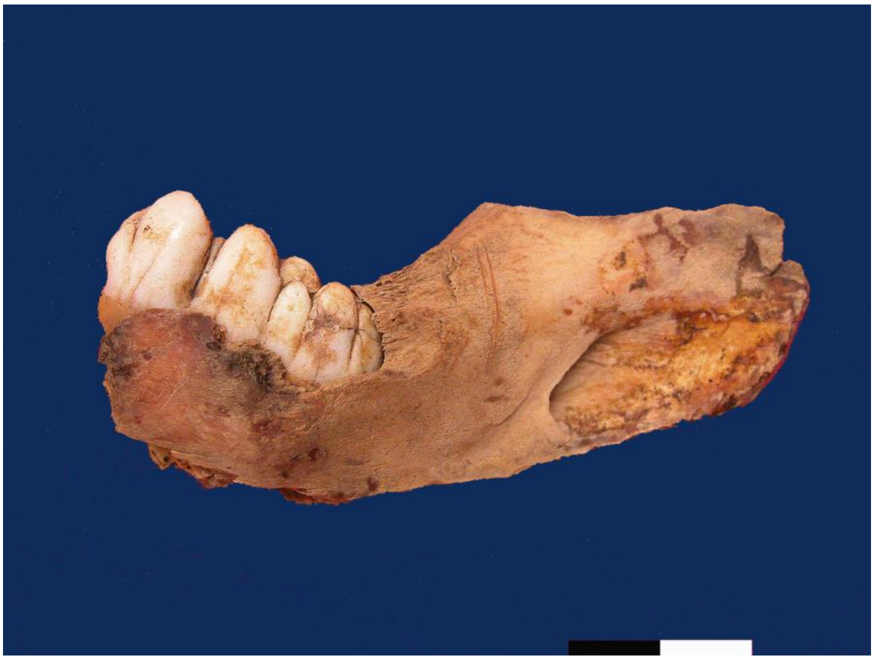

Photo 3. Jaw of Sus scrofa with fleshing signals from La Cueva de Las Palomas (Icod de los Vinos, Tenerife) (© C. del Arco).

Gran Canaria

The island of Gran Canaria conserves very important villages built with stones or opened in the rock by aboriginal people who inhabited the island from North of Africa. Examples of these are the archaeological sites of Acusa (Artenara) and Guayadeque (Agüimes- Ingenio), which are large permanent villages of natural and, mainly, man-made caves that include dwellings, communal granaries and cemeteries. In both cases, they were located next to regions suitable for agricultural activities and livestock farming.

Between the end of 19th century and the first decades of 20th century, the Canarian Museum carried out archaeological excavations in several collective funeral caves in Acusa and Guayadeque and recovered the mummies that are analysed in this article with inventory number 5 (Acusa), 10, 11 and 12 (Guayadeque). They were wrapped up in leather and vegetal fiber shrouds, a very common practice among ancient canarian settlements. The preservation of these organic materials is excellent because they were deposited in caves that keep constant temperature and humidity conditions.

Samples of one mummy from Guayadeque and of one mummy from Acusa were radiocarbon dated in 1957. The resulting dates were 536-720 cal. AD (CALIB. Radiocarbon Calibration, Execute version 7.0 html) and cal. 556-729 cal. AD (CALIB. Radiocarbon Calibration, Execute version 7.0 html) respectively. Today, is not possible to know which of the several mummies recovered from those cemeteries were selected for radiocarbon dating.

The infant mummy from Arguineguín was recovered from a funerary cave located in a ravine at the south of the island, in 1855. It was part of a private collection (Vega Grande Count collection) until it was donated to the Canarian Museum in 1901. Nowadays it is not possible to locate this funeral cave. The description of the cave was published in a local newspaper according to which the cave housed several individuals, some of them mummified and covered by leather and vegetal fiber shrouds [30].

Several photos of mummy samples from sites in Gran Canaria are provided below.

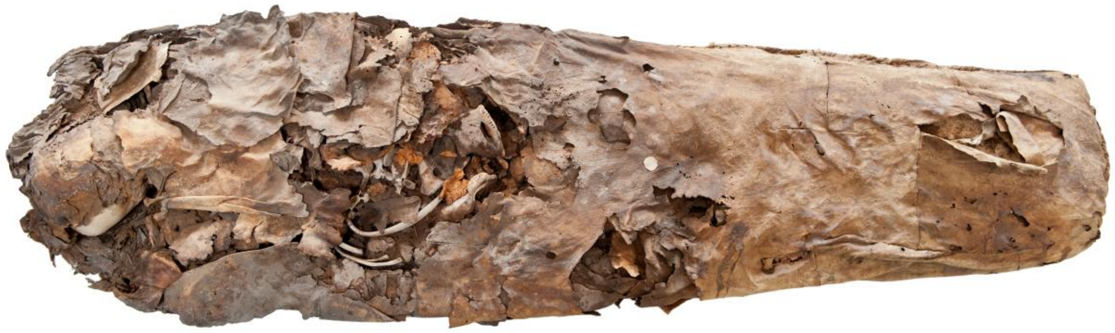

Photo 1. Juvenile mummy recovered from a collective funeral cave in Guayadeque ravin (Agüimes-Ingenio, Gran Canaria). Inventory number 11. Canarian Museum Archive.

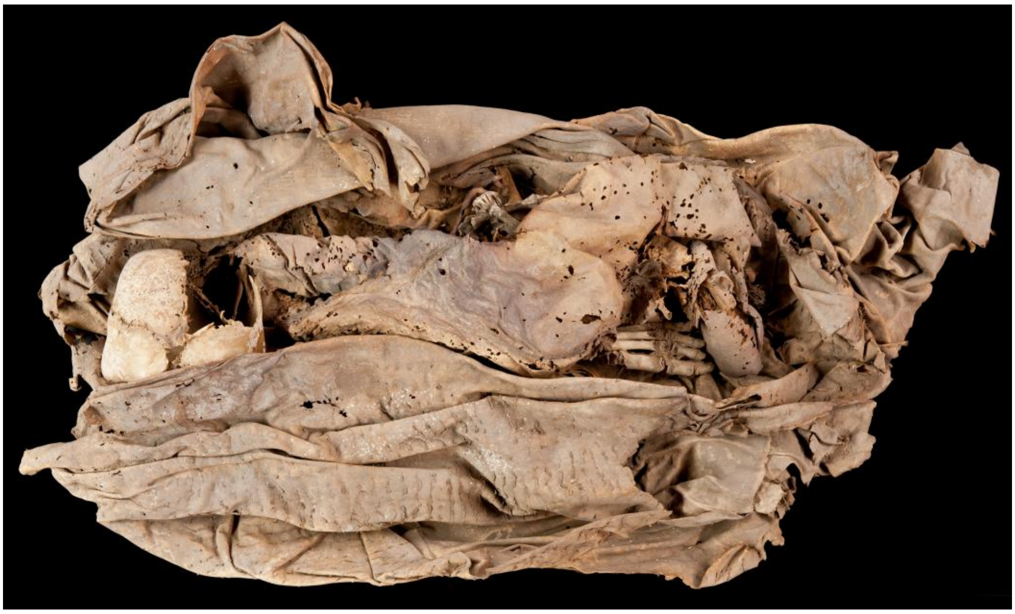

Photo 2. Infant mummy recovered from a collective funeral cave in Arguineguín (Gran Canaria). Inventory number 16. Canarian Museum Archive.

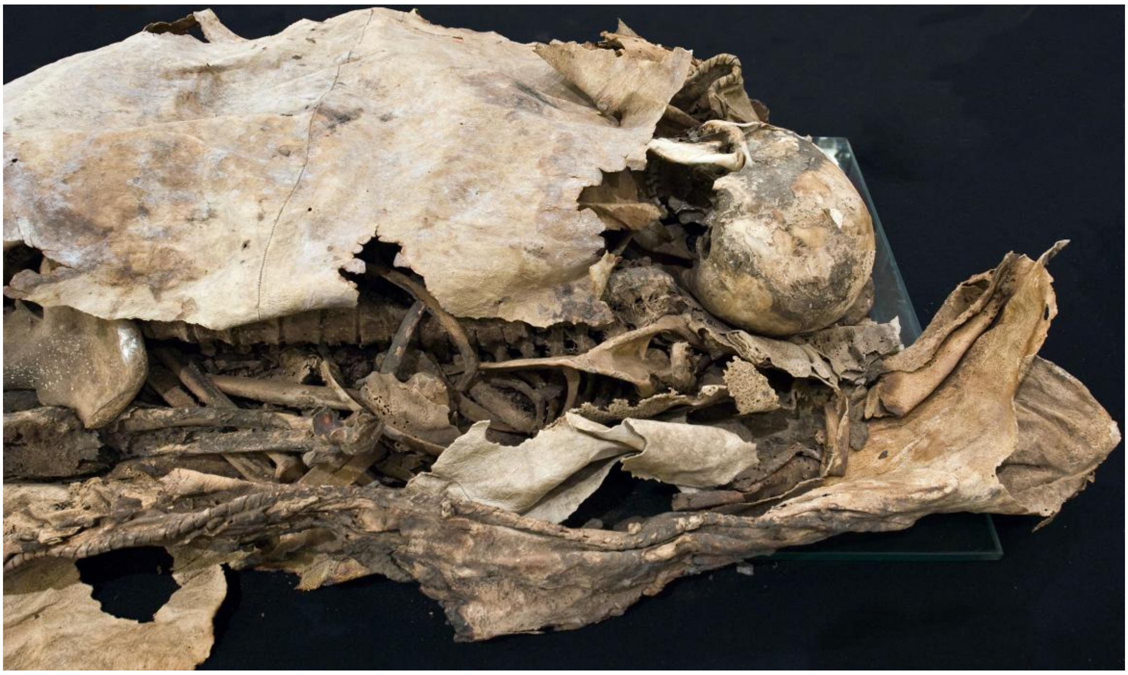

Photo 3. Mummy recovered from a collective funeral cave in Guayadeque ravin (Agüimes- Ingenio, Gran Canaria). Inventory number 12. Canarian Museum Archive.

Lanzarote

Lanzarote samples were taken under the project HAR2009-08519, “Canarias:colonitzación humana protohistórica, bioadaptación insular y transformación medioambiental” funded by Ministerio de Ciencia e Innovación, Spain [31-36].
