# Supplementary material for: Ancient DNA sheds light on the ancestry of pre-hispanic Canarian pigs
Source: Genet Sel Evol. 2015 May 6;47(1):40. doi: 10.1186/s12711-015-0115-7 (PMC4421913; doi:10.1186/s12711-015-0115-7)
Supplement: Additional file 2: Table S1. — Archaeological sites and age of Canarian pig samples used. This Table provides the ID, lab codes, archeological site and age of the Canarian pig ancient samples from which cytochrome B haplogroups (Fragment 1) and haplotypes (Fragment 2) were retrieved. List of primers used for amplification of the two 89 and 77 bp sequences of the MT-CYB gene. Table S2. provides the names and sequences of the primers used to amplify the two 89 and 77 bp sequences of the MT-CYB gene. Primers were designed with the Primer 3 software (http://bioinfo.ut.ee/primer3-0.4.0/). [file 12711_2015_115_MOESM2_ESM.pdf]

Table S1. Archaeological sites and antiquity of Canarian pig samples (ID and lab code are provided) from which cytochrome B haplogroups (Fragment 1) and haplotypes (Fragment 2) have been retrieved.

Id	Code	Site	Century	Frag 1	Frag 2
1	Buenavista1	Buenavista (Lanzarote)	10-6th BC	E1	H1
2	Buenavista2	Buenavista (Lanzarote)	4th BC	E1	H1
3	Buenavista3	Buenavista (Lanzarote)	2th BC-3th AD	-	
4	Lanzarote12	El Bebedero (Lanzarote)	4- 5th AD	E1	-
5	Palma10	Cueva del Tendal (La Palma)	9th AD	E1	H1
6	Palma11	Cueva del Tendal (La Palma)	3th AD	E1	H1
7	LaGuancha1	Hoya Brunco (Tenerife)	11th AD	E1	
8	LaGuancha3	Hoya Brunco (Tenerife)	11th AD	E1	
9	Retamar4	El Retamar (Tenerife)	15th AD	E1	
10	Paloma2	Cueva de las Palomas (Tenerife)	10th AD	E1	H1
11	Paloma4	Cueva de las Palomas (Tenerife)	2th BC	E1	H1
12	Guanches7	Cueva de los Guanches (Tenerife)	4th AD	E1	H1
13	Cabezazo1	Cueva de los Cabezazos (Tenerife)	8th AD	E1	H1
14	Cabezazo4	Cueva de los Cabezazos (Tenerife)	8th AD	E1	
15	Cabezazo6	Cueva de los Cabezazos (Tenerife)	8th AD	E1	
16	Cabezazo7	Cueva de los Cabezazos (Tenerife)	8th AD	-	
17	Guadayeque10	Guadayeque (Gran Canaria)	6-7th AD	E1	
18	Guadayeque11/1	Guadayeque (Gran Canaria)	-	E1	
19	Guadayeque11/3	Guadayeque (Gran Canaria)	-	E1	-
20	Guadayeque12	Guadayeque (Gran Canaria)	-	E1	
21	Arguineguin16	Arguineguín (Gran Canaria)	-	E1	
22	Acusa5/1	Acusa (Gran Canaria)	7-8th AD	E1	
23	Acusa5/2	Acusa (Gran Canaria)	7-8th AD	E1	

Table S2.Primers used for amplification of two fragments of 89 and 77 base pairs (bp) of the mtDNAcyt-b gene. The primers were designed with the aid of the Primer 3 software (http://bioinfo.ut.ee/primer3-0.4.0/).

**Primer Name**	**Sequence (5´-3´)**	**Product size**
CytBSus15035L/CytBSus15082H	CCTAATAAACTAGGTGGAGT/ CCTCGTTGTTTGGATGTGTGT	89 bp
CytBSus15645L/CytBSus15681H	TTCTCATCAGTTACACACAT/ GATGCTCCGTTTGCATGTAG	77 bp
