# Supplementary material for: Ancient DNA sheds light on the ancestry of pre-hispanic Canarian pigs
Source: Genet Sel Evol. 2015 May 6;47(1):40. doi: 10.1186/s12711-015-0115-7 (PMC4421913; doi:10.1186/s12711-015-0115-7)
Supplement: Additional file 3: Figure S1. — Alignment of the 48-bp sequences (PCR1) of the MT-CYB gene fragment 1 obtained from DNA from 21 Canarian pig ancient samples. Description: The diagnostic SNPs that are located at positions 15036, 15038, 15041, 15044 and 15045 (indicated in red) differentiate European: E1 (GenBankID: KJ746666), E2 (EU531827) and E4 (GU211924) haplogroups and, also, Far Asian: A1 (KP257599), A2 (KM215171), A3 (AB015072), A4 (KJ746664) and A5 (GU135825) haplogroups. Figure S2. Alignment of the 37-bp sequences (PCR2) of the MT-CYB gene fragment 1 obtained from DNA from 8 Canarian pig ancient samples. Description: The two diagnostic SNPs (indicated in red) differentiate haplotypes of haplogroup E1 present in wild boar individuals from the Near East: H12 (GenBank ID: EU531818), H31 (EU531827), H52 (EU531832), H53 (EU531833), and H54 (EU531834); and from Europe: H1 (AY237496), H4 (AY237512), H15 (AB015072), H16 (EU531821), H17 (EF061501), H18 (AY237516), H24 (AF136542), H27 (EU531824), H32 (EU531828), H34 (EU531830), H65 (AM492593) and H66 (AM492620). [file 12711_2015_115_MOESM3_ESM.pdf]

Figure S1. Alignment of a 48 bp corresponding to the mtDNAcyt-b gene fragment 1 obtained in the 21 ancient samples of Canarian pigs. In red the diagnostic SNPs (located at positions 15036, 15038, 15041, 15044 and 15045) that allow the differentiation amongst European: E1 (GenBankID: KJ746666), E2 (EU531827) and E4 (GU211924); and Far Asian: A1 (KP257599), A2 (KM215171), A3 (AB015072), A4 (KJ746664) and A5 (GU135825) haplogroups.

```
>E1      CCTAATAAACTAGGTGGAGTGTTGGCCCTAGTAGCCTCCATCCTAATCCTAATTTTAATGCCCATACTACACACATCCAAACAACGAGG
>E2                          ......T.........................................
>E4                          ..........A.....................................
>A1                          .C.A..T...A.....................................
>A2                          .C.A..T.........................................
>A3                          ...A............................................
>A4                          AC.A..T...A.....................................
>A5                          ...A..T.........................................
```

Buenavista1 (Buenavista, Lanzarote)

```
>HaplogrupoE1   CCTAATAAACTAGGTGGAGTGTTGGCCCTAGTAGCCTCCATCCTAATCCTAATTTTAATGCCCATACTACACACATCCAAACAACGAGG
>PCR1_1                             .....T..........................................
>PCR1_2                             .................N..............................
>PCR1_3                             ...........................N....................
>PCR1_4                             .....................N..........................
>PCR1_5                             ................................................
>PCR1_6                             ................................................
>PCR1_7                             ................................................
>PCR1_8                             ................................................
```

Buenavista1 (replicated in an independent laboratory, UPF)

```
>HaplogrupoE1   CCTAATAAACTAGGTGGAGTGTTGGCCCTAGTAGCCTCCATCCTAATCCTAATTTTAATGCCCATACTACACACATCCAAACAACGAGG
>PCR1_1                             ................................................
>PCR1_2                             ................................................
>PCR1_3                             ................................................
>PCR1_4                             ................................................
>PCR1_5                             ................................................
>PCR1_6                             ................................................
>PCR1_7                             ................................................
>PCR1_8                             ................................................
```

Buenavista2 (Buenavista, Lanzarote)

```
>HaplogrupoE1   CCTAATAAACTAGGTGGAGTGTTGGCCCTAGTAGCCTCCATCCTAATCCTAATTTTAATGCCCATACTACACACATCCAAACAACGAGG
>PCR1_1                             .......T........................................
>PCR1_2                             .......T...........N............................
>PCR1_3                             ..........................N.....................
>PCR1_4                             ...................N............................
>PCR1_5                             ............................N...................
```

Lanzarote 12 (El Bebedero, Lanzarote)

```
>HaplogrupoE1   CCTAATAAACTAGGTGGAGTGTTGGCCCTAGTAGCCTCCATCCTAATCCTAATTTTAATGCCCATACTACACACATCCAAACAACGAGG
>PCR1_1                             .....................T..........................
>PCR1_2                             .....................T..........................
>PCR1_3                             ................................................
>PCR1_4                             .....................T..........................
>PCR1_5                             .....................TN.....N...................
>PCR1_6                             .....................T..........................
>PCR1_7                             .....................T..........................
>PCR1_8                             .....................T..........................
>HaplogrupoE1   CCTAATAAACTAGGTGGAGTGTTGGCCCTAGTAGCCTCCATCCTAATCCTAATTTTAATGCCCATACTACACACATCCAAACAACGAGG
>PCR2_1                             .....................T..........................
>PCR2_2                             ................N....T..........................
>PCR2_3                             ................C..N.T..........................
>PCR2_4                             ...................N.T..........................
```

Palma10 (Cueva del Tendal, La Palma)

```
>HaplogrupoE1   CCTAATAAACTAGGTGGAGTGTTGGCCCTAGTAGCCTCCATCCTAATCCTAATTTTAATGCCCATACTACACACATCCAAACAACGAGG
>PCR1_1                             ................................................
>PCR1_2                             ......................T....TT...................
>PCR1_3                             ...........................T....................
```

```
>PCR1_4                     ................................................
>PCR1_5                     ................................................
>PCR1_6                     .....TTT.............T..........................
>PCR1_7                     ................................................
>PCR1_8                     .....T..........................................
>HaplogrupoE1     CCTAATAAACTAGGTGGAGTGTTGGCCCTAGTAGCCTCCATCCTAATCCTAATTTTAATGCCCATACTACACACATCCAAACAACGAGG
>PCR2_1                     ................C...............................
>PCR2_2                     ................................................
>PCR2_3                     ................................................
>PCR2_4                     .....................N...N......................
```

Palma11 (Cueva del Tendal, La Palma)

```
>HaplogrupoE1     CCTAATAAACTAGGTGGAGTGTTGGCCCTAGTAGCCTCCATCCTAATCCTAATTTTAATGCCCATACTACACACATCCAAACAACGAGG
>PCR1_1                     .................N.........NN...................
>PCR1_2                     ..............T.............T...................
>PCR1_3                     ...A............................................
>PCR1_4                     ................................................
>PCR1_5                     ................................................
>PCR1_6                     ...........................NT...................
>PCR1_7                     ..................T...T.....T...................
>PCR1_8                     ......TT..............N....TN...........T.......
>PCR1_9                     ......TT......TT.TT..TT....TT...................
>PCR1_10                    .....T...........T...T..........................
>PCR1_11                    .....T..........................................
```

LaGuancha1 (Hoya Bruco, Tenerife)

```
>HaplogrupoE1     CCTAATAAACTAGGTGGAGTGTTGGCCCTAGTAGCCTCCATCCTAATCCTAATTTTAATGCCCATACTACACACATCCAAACAACGAGG
>PCR1_1                     ................................................
>PCR1_2                     ................N...............................
>PCR1_3                     ................N.NN............................
>PCR1_4                     ................N...............................
>PCR1_5                     ................N...............................
>PCR1_6                     ......................NN........................
>PCR1_7                     ......................N.........................
>PCR1_8                     ......................N.........................
>HaplogrupoE1     CCTAATAAACTAGGTGGAGTGTTGGCCCTAGTAGCCTCCATCCTAATCCTAATTTTAATGCCCATACTACACACATCCAAACAACGAGG
>PCR2_1                     ................................................
>PCR2_2                     ...........................T....................
>PCR2_3                     ................................................
>PCR2_4                     ................................................
>PCR1_5                     ................................................
>PCR1_6                     ............................T...................
>PCR1_7                     ................................................
>PCR1_8                     ..................T.............................
```

LaGuancha3 (Hoya Bruco, Tenerife)

```
>HaplogrupoE1     CCTAATAAACTAGGTGGAGTGTTGGCCCTAGTAGCCTCCATCCTAATCCTAATTTTAATGCCCATACTACACACATCCAAACAACGAGG
>PCR1_1                     ................N...............................
>PCR1_2                     ................N...............................
>PCR1_3                     ................................................
>PCR1_4                     ................................................
>PCR1_5                     ................................................
>PCR1_6                     ................................................
>PCR1_7                     ......................N.........................
```

Retamar4 (El Retamar, Tenerife)

```
>HaplogrupoE1     CCTAATAAACTAGGTGGAGTGTTGGCCCTAGTAGCCTCCATCCTAATCCTAATTTTAATGCCCATACTACACACATCCAAACAACGAGG
>PCR1_1                     ....................................G...........
>PCR1_2                     ................................................
>PCR1_3                     ................................................
>PCR1_4                     ................................................
>PCR1_5                     ................................................
>PCR1_6                     ................................................
>PCR1_7                     ................C...............................
>PCR1_8                     ................................................
>HaplogrupoE1     CCTAATAAACTAGGTGGAGTGTTGGCCCTAGTAGCCTCCATCCTAATCCTAATTTTAATGCCCATACTACACACATCCAAACAACGAGG
>PCR2_1                     ................................................
>PCR2_2                     ................................................
>PCR2_3                     ................................................
>PCR2_4                     ................................................
>PCR1_5                     ................................................
>PCR1_6                     ................................................
```

Paloma2 (Cueva de las Palomas, Tenerife)

```
>HaplogrupoE1     CCTAATAAACTAGGTGGAGTGTTGGCCCTAGTAGCCTCCATCCTAATCCTAATTTTAATGCCCATACTACACACATCCAAACAACGAGG
>PCR1_1                     ................................................
>PCR1_2                     ................................................
>PCR1_3                     ................................................
>PCR1_4                     .....T..........................................
>PCR1_5                     ................N...............................
>PCR1_6                     .......N........................................
>PCR1_7                     ..................T.............................
```

```
>PCR1_8                                 ................................................
>HaplogrupoE1       CCTAATAAACTAGGTGGAGTGTTGGCCCTAGTAGCCTCCATCCTAATCCTAATTTTAATGCCCATACTACACACATCCAAACAACGAGG
>PCR2_1                                 ..............T.................................
>PCR2_2                                 .....................T..........................
>PCR2_3                                 ......................T.........................
>PCR2_4                                 ........................................T.......
>PCR2_5                                 ................................................
```

Paloma4 (Cueva de las Palomas, Tenerife)

```
>HaplogrupoE1       CCTAATAAACTAGGTGGAGTGTTGGCCCTAGTAGCCTCCATCCTAATCCTAATTTTAATGCCCATACTACACACATCCAAACAACGAGG
>PCR1_1                                 ................................................
>PCR1_2                                 ..........A.....................................
>PCR1_3                                 ................................................
>PCR1_4                                 ..........A.....................................
>PCR1_5                                 ................................................
>PCR1_6                                 ................................................
>PCR1_7                                 ..................N...N.........................
>PCR1_8                                 .................................N..N...........
>HaplogrupoE1       CCTAATAAACTAGGTGGAGTGTTGGCCCTAGTAGCCTCCATCCTAATCCTAATTTTAATGCCCATACTACACACATCCAAACAACGAGG
>PCR2_1                                 .....T.........T.NC..TT....N....................
>PCR2_2                                 ...............T................................
>PCR2_3                                 ............................T...................
>PCR2_4                                 .....................TT.....T...................
>PCR2_5                                 .................T..........T...................
>PCR2_6                                 .....T.T......TT.....TT....TT...........T.......
```

Guanches7 (Cueva de los Guanches, Tenerife)

```
>HaplogrupoE1       CCTAATAAACTAGGTGGAGTGTTGGCCCTAGTAGCCTCCATCCTAATCCTAATTTTAATGCCCATACTACACACATCCAAACAACGAGG
>PCR1_1                                 ..................T.............................
>PCR1_2                                 ................................................
>PCR1_3                                 .....................T..........................
>PCR1_4                                 .......................................A........
>PCR1_5                                 ..............TT............T...................
>PCR1_6                                 ................................................
>PCR1_7                                 ..................T........T....................
>PCR1_8                                 .....................N..........................
>PCR1_9                                 .....TT.......TT..T.............................
>PCR1_10                                .....TT........T..T........TT...................
>PCR1_11                                .....................T..........................
>PCR1_12                                ..............N.......N.........................
>PCR1_13                                ..............T..T.........T....................
>PCR1_14                                .......................................A........
```

Cabezazo1 (Cueva de los Cabezazos, Tenerife)

```
>HaplogrupoE1       CCTAATAAACTAGGTGGAGTGTTGGCCCTAGTAGCCTCCATCCTAATCCTAATTTTAATGCCCATACTACACACATCCAAACAACGAGG
>PCR1_1                                 ......T......T.............T...................
>PCR1_2                                 ......T.........T..............................
>PCR1_3                                 .....................T.........................
>PCR1_4                                 ....................TT.........................
>PCR1_5                                 .............T.................................
>PCR1_6                                 .....T..........T..........T...................
>PCR1_7                                 .............N......NN.........................
>PCR1_8                                 ..............T.T...T..........................
>PCR1_9                                 ...............................................
>PCR1_10                                ...............................................
>PCR1_11                                .............T.N.T..T.....T............T.......
```

Cabezazo4 (Cueva de los Cabezazos, Tenerife)

```
>HaplogrupoE1       CCTAATAAACTAGGTGGAGTGTTGGCCCTAGTAGCCTCCATCCTAATCCTAATTTTAATGCCCATACTACACACATCCAAACAACGAGG
>PCR1_1                                 ................................................
>PCR1_2                                 ................................................
>PCR1_3                                 ...AA.....A.....................................
>PCR1_4                                 ..............TT......T.........................
>PCR1_5                                 ......TT......T......TT.........................
>PCR1_6                                 ...............T.T...T.....T....................
>PCR1_7                                 ................................................
>PCR1_8                                 .......T......TT.T....T.........................
>PCR1_9                                 ..............T......TT.................T.......
>PCR1_10                                ...............N..N..NN.........................
>PCR1_11                                .....TNN.........T....N.....N...................
>PCR1_12                                ..............TT..T..TT.........................
>PCR1_13                                ......N........N.N.........N....................
>PCR1_14                                ...............N.....NN....N....................
>PCR1_15                                ..............T..T.........T....................
>PCR1_16                                .....T..........NT....T.........................
```

Cabezazo6 (Cueva de los Cabezazos, Tenerife)

```
>HaplogrupoE1       CCTAATAAACTAGGTGGAGTGTTGGCCCTAGTAGCCTCCATCCTAATCCTAATTTTAATGCCCATACTACACACATCCAAACAACGAGG
>PCR1_1                                 .......T.............T.....TT...................
>PCR1_2                                 ...........................TT...................
>PCR1_3                                 ..............NN.....N......T...................
```

```
>PCR1_4                                 .....T.T...................T....................
>PCR1_5                                 .......................................A........
>PCR1_6                                 .....TNN.......N.N...T......N...................
>PCR1_7                                 .....TTT..........T.............................
>PCR1_8                                 ................................................
>HaplogrupoE1       CCTAATAAACTAGGTGGAGTGTTGGCCCTAGTAGCCTCCATCCTAATCCTAATTTTAATGCCCATACTACACACATCCAAACAACGAGG
>PCR2_1                                 ....A.....A.....................................
>PCR2_2                                 ..........A.....................................
>PCR2_3                                 ..........A.....................................
>PCR2_4                                 ................................................
>PCR1_5                                 ................................................
```

Guadayeque10 (Guadayeque, Gran Canaria)

```
>HaplogrupoE1       CCTAATAAACTAGGTGGAGTGTTGGCCCTAGTAGCCTCCATCCTAATCCTAATTTTAATGCCCATACTACACACATCCAAACAACGAGG
>PCR1_1                                 .................N..NT..........................
>PCR1_2                                 .................NT.............................
>PCR1_3                                 ..................T.............................
>PCR1_4                                 ..................T.............................
>PCR1_5                                 ..................T.............................
>PCR1_6                                 ..................T.............................
>PCR1_7                                 ......................N........................
>PCR1_8                                 ..................T...N.........................
```

Guadayeque11/1 (Guadayeque, Gran Canaria)

```
>HaplogrupoE1       CCTAATAAACTAGGTGGAGTGTTGGCCCTAGTAGCCTCCATCCTAATCCTAATTTTAATGCCCATACTACACACATCCAAACAACGAGG
>PCR1_1                                 .....................T..........................
>PCR1_2                                 ................................................
>PCR1_3                                 .....................T.....T............T.......
>PCR1_4                                 ..................T..T.....T............T.......
>PCR1_5                                 ................................................
>PCR1_6                                 .....................T..........................
>PCR1_7                                 ................................................
```

Guadayeque11/3 (Guadayeque, Gran Canaria)

```
>HaplogrupoE1       CCTAATAAACTAGGTGGAGTGTTGGCCCTAGTAGCCTCCATCCTAATCCTAATTTTAATGCCCATACTACACACATCCAAACAACGAGG
>PCR1_1                                 ................................................
>PCR1_2                                 ................................................
>PCR1_3                                 ................................................
>PCR1_4                                 ................................................
>PCR1_5                                 ................................................
>PCR1_6                                 ................................................
>PCR1_7                                 ................................................
>PCR1_8                                 ................................................
>HaplogrupoE1       CCTAATAAACTAGGTGGAGTGTTGGCCCTAGTAGCCTCCATCCTAATCCTAATTTTAATGCCCATACTACACACATCCAAACAACGAGG
>PCR2_1                                 ................................................
>PCR2_2                                 ................................................
>PCR2_3                                 ................................................
>PCR2_4                                 ................................................
>PCR1_5                                 ................................................
>PCR1_6                                 ................................................
>PCR1_7                                 ................................................
```

Guadayeque12 (Guadayeque, Gran Canaria)

```
>HaplogrupoE1       CCTAATAAACTAGGTGGAGTGTTGGCCCTAGTAGCCTCCATCCTAATCCTAATTTTAATGCCCATACTACACACATCCAAACAACGAGG
>PCR1_1                                 ...............T..N........TT...................
>PCR1_2                                 ...............T..N...N....TT...................
>PCR1_3                                 ...............T..T........TT...................
>PCR1_4                                 .......T.............TT....T............T.......
>PCR1_5                                 .......T.............TNN.N.T............T.......
>PCR1_6                                 .......T.............TTN...T............T.......
>PCR1_7                                 . ...............T..T........TT...................
```

Arguineguin16 (Arguineguin, Gran Canaria)

```
>HaplogrupoE1       CCTAATAAACTAGGTGGAGTGTTGGCCCTAGTAGCCTCCATCCTAATCCTAATTTTAATGCCCATACTACACACATCCAAACAACGAGG
>PCR1_1                                 ................................................
>PCR1_2                                 ................................................
>PCR1_3                                 ................................................
>PCR1_4                                 ................................................
>PCR1_5                                 ................................................
>PCR1_6                                 ................................................
>PCR1_7                                 ................................................
>PCR1_8                                 ................................................
>PCR1_9                                 ................................................
>PCR1_10                                ................................................
>PCR1_11                                ................................................
```

Acusa 5/1 (Acusa,Gran Canaria)

```
>HaplogrupoE1       CCTAATAAACTAGGTGGAGTGTTGGCCCTAGTAGCCTCCATCCTAATCCTAATTTTAATGCCCATACTACACACATCCAAACAACGAGG
>PCR1_1                                 .....................T..........................
>PCR1_2                                 ......................N.........................
```

```
>PCR1_3                                     ......................N.........................
>PCR1_4                                     .....................T..........................
>PCR1_5                                     ................................................
>PCR1_6                                     ................................................
>HaplogrupoE1       CCTAATAAACTAGGTGGAGTGTTGGCCCTAGTAGCCTCCATCCTAATCCTAATTTTAATGCCCATACTACACACATCCAAACAACGAGG
>PCR2_1                                     ...............N................................
>PCR2_2                                     ...............N.N..............................
>PCR2_3                                     ...............N.N..............................
>PCR2_4                                     ...............N................................
>PCR1_5                                     ................................................
>PCR1_6                                     ................................................
>PCR1_7                                     ................................................
```

Acusa 5/2 (Acusa,Gran Canaria)

```
>HaplogrupoE1       CCTAATAAACTAGGTGGAGTGTTGGCCCTAGTAGCCTCCATCCTAATCCTAATTTTAATGCCCATACTACACACATCCAAACAACGAGG
>PCR1_1                                     .................T...T..........C...............
>PCR1_2                                     ................................................
>PCR1_3                                     ..........................N....N................
>PCR1_4                                     ...............................N................
>PCR1_5                                     ..........................N.....................
>PCR1_6                                     ..........................N.....................
>PCR1_7                                     ................................................
```

Figure S2. Alignment of a 37 bp corresponding to the mtDNAcyt-b gene fragment 2 obtained in the 8 ancient samples of Canarian pigs. In red the two diagnostic SNPs that allow the differentiation amongst haplotypes of the haplogroup E1 present in wild boar individuals from Near East: H12 (GenBankID: EU531818), H31 (EU531827), H52 (EU531832), H53 (EU531833), and H54 (EU531834); and from Europe: H1 (AY237496), H4 (AY237512), H15 (AB015072), H16 (EU531821), H17 (EF061501), H18 (AY237516), H24 (AF136542), H27 (EU531824), H32 (EU531828), H34 (EU531830), H65 (AM492593) and H66 (AM492620) haplogroups.

```
>H1EWB  TTCTCATCAGTTACACACATTTGTCGAGACGTAAATTACGGATGAGTTATTCGCTATCTACATGCAAACGGAGCATC
>H12NE                      C...................................C
>H54NE                      C........................A..........C
>H53                        C........................A..........C
>H52                        C........................A..........C
>H31                        C...................................C
>H15                        .....................................
>H16                        .....................................
>H65                        .....................................
>H24                        ...........................C.........
>H66                        .....................................
>H4                         .....................................
>H17                        .....................................
>H34                        .....................................
>H18                        .....................................
>H32                        .....................................
>H27                        .....................................
```

Buenavista1 (Lanzarote)

```
>H1     TTCTCATCAGTTACACACATTTGTCGAGACGTAAATTACGGATGAGTTATTCGCTATCTACATGCAAACGGAGCATC
>PCR1_1                     .....................................
>PCR1_2                     .....................................
>PCR1_3                     .....................................
>PCR1_4                     .....................................
>PCR1_5                     .....................................
>PCR1_6                     .....................................
>PCR1_7                     .....................................
>PCR1_8                     .....................................
>H1     TTCTCATCAGTTACACACATTTGTCGAGACGTAAATTACGGATGAGTTATTCGCTATCTACATGCAAACGGAGCATC
>PCR2_1                     .....................................
>PCR2_2                     .....................................
>PCR2_3                     .....................................
>PCR2_4                     .....................................
>PCR2_5                     .....................................
>PCR2_6                     .....................................
>PCR2_7                     .....................................
```

Buenavista1 (replicated in an independent laboratory, UPF)

```
>H1    TTCTCATCAGTTACACACATTTGTCGAGACGTAAATTACGGATGAGTTATTCGCTATCTACATGCAAACGGAGCATC
>PCR1_1                    .....................................
>PCR1_2                    .....................................
>PCR1_3                    .....................................
>PCR1_4                    .....................................
>PCR1_5                    .....................................
>PCR1_6                    .....................................
>PCR1_7                    .....................................
>PCR1_8                    .....................................
```

Buenavista2 (Lanzarote)

```
>H1    TTCTCATCAGTTACACACATTTGTCGAGACGTAAATTACGGATGAGTTATTCGCTATCTACATGCAAACGGAGCATC
>PCR1_1                    .....................................
>PCR1_2                    ..................T..................
>PCR1_3                    .....................................
>PCR1_4                    .....................................
>PCR1_5                    .....................................
>PCR1_6                    .....................................
>PCR1_7                    .....................................
>PCR1_8                    .....................................
>H1    TTCTCATCAGTTACACACATTTGTCGAGACGTAAATTACGGATGAGTTATTCGCTATCTACATGCAAACGGAGCATC
>PCR2_1                    .....................................
>PCR2_2                    .....................................
>PCR2_3                    .....................................
>PCR2_4                    .....................................
>PCR2_5                    .....................................
>PCR2_6                    .....................................
>PCR2_7                    .....................................
```

Palma10 (La Palma)

```
>H1    TTCTCATCAGTTACACACATTTGTCGAGACGTAAATTACGGATGAGTTATTCGCTATCTACATGCAAACGGAGCATC
>PCR1_1                    .....................................
>PCR1_2                    ..............G......................
>PCR1_3                    .....................................
>PCR1_4                    ...................AA....A...........
>PCR1_5                    .....................................
>PCR1_6                    .........................A...........
>PCR1_7                    .....................................
>PCR1_8                    .........................A...........
```

Palma11 (La Palma)

```
>H1    TTCTCATCAGTTACACACATTTGTCGAGACGTAAATTACGGATGAGTTATTCGCTATCTACATGCAAACGGAGCATC
>PCR1_1                    ..A.......A..........................
>PCR1_2                    .....................................
>PCR1_3                    .................................T...
>PCR1_4                    ................................AT...
>PCR1_5                    .....................................
>PCR1_6                    .....................................
>PCR1_7                    .....................................
>PCR1_8                    .....................................
```

Paloma2 (Tenerife)

```
>H1    TTCTCATCAGTTACACACATTTGTCGAGACGTAAATTACGGATGAGTTATTCGCTATCTACATGCAAACGGAGCATC
>PCR1_1                    .....................................
>PCR1_2                    .....................................
>PCR1_3                    .....................................
>PCR1_4                    .....................................
>PCR1_5                    .....................................
>PCR1_6                    .....................................
>PCR1_7                    .....................................
>PCR1_8                    .....................................
```

Paloma4 (Tenerife)

```
>H1      TTCTCATCAGTTACACACATTTGTCGAGACGTAAATTACGGATGAGTTATTCGCTATCTACATGCAAACGGAGCATC
>PCR1_1                      .........................A...........
>PCR1_2                      .................................T...
>PCR1_3                      .....................................
>PCR1_4                      .....................................
>PCR1_5                      .....................................
>PCR1_6                      .....................................
>PCR1_7                      .....................................
>PCR1_8                      .....................................
```

Cabezazo1 (Tenerife)

```
>H1      TTCTCATCAGTTACACACATTTGTCGAGACGTAAATTACGGATGAGTTATTCGCTATCTACATGCAAACGGAGCATC
>PCR1_1                      .......A............A...........A....
>PCR1_2                      .......A............A...........A....
>PCR1_3                      .....................................
>PCR1_4                      .....................................
>PCR1_5                      .....................................
>PCR1_6                      .....................................
>PCR1_7                      .....................................
>PCR1_8                      .....................................
>H1      TTCTCATCAGTTACACACATTTGTCGAGACGTAAATTACGGATGAGTTATTCGCTATCTACATGCAAACGGAGCATC
>PCR2_1                      .....................................
>PCR2_2                      .....................................
>PCR2_3                      .....................................
>PCR2_4                      ....................................
```

Guanche7 (Tenerife)

```
>H1      TTCTCATCAGTTACACACATTTGTCGAGACGTAAATTACGGATGAGTTATTCGCTATCTACATGCAAACGGAGCATC
>PCR1_1                      .....................................
>PCR1_2                      .....................................
>PCR1_3                      ..................T..................
>PCR1_4                      ..................T..................
>PCR1_5                      .....................................
>PCR1_6                      .....................................
>PCR1_7                      .........................A...........
>PCR1_8                      .........T...........................
>H1      TTCTCATCAGTTACACACATTTGTCGAGACGTAAATTACGGATGAGTTATTCGCTATCTACATGCAAACGGAGCATC
>PCR2_1                      .....................................
>PCR2_2                      .....................................
>PCR2_3                      .....................................
>PCR2_4                      .....................................
```
